# Prevalence of Atrial Fibrillation and Associated Mortality Among Hospitalized Patients With COVID-19: A Systematic Review and Meta-Analysis

**DOI:** 10.3389/fcvm.2021.720129

**Published:** 2021-10-13

**Authors:** Zuwei Li, Wen Shao, Jing Zhang, Jianyong Ma, Shanshan Huang, Peng Yu, Wengen Zhu, Xiao Liu

**Affiliations:** ^1^Cardiology Department, The Affiliated Hospital of Jiangxi University of Chinese Medicine, Nanchang, China; ^2^Endocrine Department, The Second Affiliated Hospital of Nanchang University, Nanchang, China; ^3^Anesthesiology Department, The Second Affiliated Hospital of Nanchang University, Nanchang, China; ^4^Department of Pharmacology and Systems Physiology, University of Cincinnati College of Medicine, Cincinnati, OH, United States; ^5^Department of Cardiology, The First Affiliated Hospital of Sun Yat-sen University, Guangzhou, China; ^6^Cardiology Department, The Sun Yat-sen Memorial Hospital of Sun Yat-sen University, Guangzhou, China; ^7^Guangdong Province Key Laboratory of Arrhythmia and Electrophysiology, Guangzhou, China

**Keywords:** atrial fibrillation, COVID-19, death, prevalence, meta-analysis

## Abstract

**Background:** Epidemiological studies have shown that atrial fibrillation (AF) is a potential cardiovascular complication of coronavirus disease 2019 (COVID-19). We aimed to perform a systematic review and meta-analysis to clarify the prevalence and clinical impact of AF and new-onset AF in patients with COVID-19.

**Methods:** PubMed, Embase, the Cochrane Library, and MedRxiv up to February 27, 2021, were searched to identify studies that reported the prevalence and clinical impact of AF and new-onset AF in patients with COVID-19. The study was registered with PROSPERO (CRD42021238423).

**Results:** Nineteen eligible studies were included with a total of 21,653 hospitalized patients. The pooled prevalence of AF was 11% in patients with COVID-19. Older (≥60 years of age) patients with COVID-19 had a nearly 2.5-fold higher prevalence of AF than younger (<60 years of age) patients with COVID-19 (13 vs. 5%). Europeans had the highest prevalence of AF (15%), followed by Americans (11%), Asians (6%), and Africans (2%). The prevalence of AF in patients with severe COVID-19 was 6-fold higher than in patients with non-severe COVID-19 (19 vs. 3%). Furthermore, AF (OR: 2.98, 95% CI: 1.91 to 4.66) and new-onset AF (OR: 2.32, 95% CI: 1.60 to 3.37) were significantly associated with an increased risk of all-cause mortality among patients with COVID-19.

**Conclusion:** AF is quite common among hospitalized patients with COVID-19, particularly among older (≥60 years of age) patients with COVID-19 and patients with severe COVID-19. Moreover, AF and new-onset AF were independently associated with an increased risk of all-cause mortality among hospitalized patients with COVID-19.

## Introduction

Severe acute respiratory syndrome coronavirus 2 (SARS-CoV-2) is the pathogen of coronavirus disease 2019 (COVID-19), which emerged in December 2019 and has since caused a global epidemic. As of November 14, 2020, over 50 million cases of COVID-19 infection have been reported worldwide, resulting in more than 1 million deaths. Previous studies have confirmed that pneumonia is not only an infectious disease affecting the respiratory system, but it also has a significant impact on the cardiovascular system, leading to heart failure, arrhythmias, and myocardial ischemia (1–3). In addition to fever as the primary symptom, there are also initial clinical manifestations of the cardiovascular system among patients with COVID-19, (4, 5) indicating that cardiovascular diseases are potential complications of COVID-19 (6, 7).

Atrial fibrillation (AF) is the most common arrhythmia and can lead to stroke, peripheral embolization, heart failure, and other unfavorable outcomes (8). The prevalence of AF is between approximately 2.3% and 3.4% in the general population (9, 10). However, for patients with pulmonary disease, critical illness, or systemic inflammatory response syndrome, the prevalence and clinical impact of AF are even more substantial (11–13).

More recently, numerous epidemiological studies have shown an increased risk of AF and new-onset AF among patients with COVID-19 but have yielded inconsistent results (14–32). Moreover, accumulating literature has demonstrated that AF or new-onset AF might be significantly associated with the worst outcomes (e.g., mortality) in patients with COVID-19 (21, 25, 29). Subsequently, several meta-analyses have examined the relationship between COVID-19 and AF (33–36). However, these studies focused on arrhythmias or AF and only examined the association between AF and pooled unfavorable outcomes among patients with COVID-19. It is not clear whether AF increases the risk of death among patients with COVID-19. Furthermore, no studies to date have assessed the prevalence and clinical impact of new-onset AF in patients with COVID-19.

To help clinicians understand the potential damage to the cardiovascular system caused by COVID-19 and strengthen the monitoring and preservation of cardiac function, we conducted a systematic review and meta-analysis of observational studies to clarify the prevalence and clinical impact of AF and new-onset AF in patients with COVID-19.

## Methods

### Protocol Registration and Search Strategy

This study was registered with PROSPERO (International prospective register of systematic reviews. https://www.crd.york.ac.uk/PROSPERO/ -registration number-CRD 42021238423). We performed this meta-analysis following the Preferred Reporting Items for Systematic Reviews and Meta-Analyses (PRISMA) statement (Supplemental Table 1) (37).

Two authors (W. L. and X. L.) independently conducted the database search, selection, data extraction, and statistical analysis. Four databases were searched for all related studies, including PubMed, Embase, the Cochrane Library, and MedRxiv (https://www.medrxiv.org/), up to February 27, 2021. No language restrictions were applied. The following search terms were used for all databases: (“2019-novel coronavirus” OR “SARS-CoV-2” OR “COVID-19” OR “2019-nCoV” OR “COVID 19” OR “severe acute respiratory syndrome coronavirus 2”) AND (“atrial fibrillation” OR “atrial fibrillations” OR “auricular fibrillation” OR “auricular fibrillations”). In addition, the conference abstracts and bibliographies of related literature were scanned to obtain other articles that might meet the requirements.

### Selection Criteria and Study Selection

Studies were included if they met the following inclusion criteria: (1) patients in the literature were adults (>18 years of age) who were diagnosed with COVID-19 according to polymerase chain reaction (PCR) tests and had sinus rhythm at admission according to a 12-lead electrocardiogram (ECG); (2) studies reported the prevalence of AF during hospital admission and/or the association between AF and outcomes (e.g., all-cause mortality) in patients with COVID-19; and (3) articles were cohort or nested case–control studies. Accordingly, studies with the following conditions were excluded: (1) reviews, meta-analyses, congress abstracts, practice guidelines, patents, cases, editorials, replies, or comments; and (2) data of the articles remained unavailable after contacting the corresponding authors for further information.

The initial search results were imported into EndNote X8.2 software (Thomson Reuters, New York, NY) for management. Subsequently, duplications were eliminated automatically and manually. First, we examined the citation titles and abstracts. After the preliminary screening, we retrieved full reports that were likely to meet the predefined inclusion criteria. Any inconsistency was resolved through discussion (W. S. and X. L.) until a consensus was reached.

### Data Collection and Quality Assessment

Data were extracted based on prespecified inclusion criteria. The following information was abstracted: study characteristics (first author's name, publication year, country, and study design), patient characteristics (sample size, age, sex, and medications), exposure (AF diagnosis and number of episodes during hospitalization), and outcomes (number of events, adjusted OR/RRs and the corresponding 95% CI, and adjustments).

For studies that reported the prevalence of AF, the Joanna Briggs Institute critical appraisal checklist was used to assess the study quality. For studies that reported the association between AF and outcomes in patients with COVID-19, the Newcastle–Ottawa quality scale (NOS) was applied. Case-control studies were appraised on selection, comparability, and exposure, while cohort studies were appraised on selection, comparability, and outcomes. Studies with an NOS of ≥6 stars were considered moderate- to high-quality articles.

### Statistical Analysis

RevMan software, version 5.3 (The Cochrane Collaboration 2014, Nordic Cochrane Center Copenhagen, Denmark) and Stata software (Version 14.0, Stata Corp LP, College Station, Texas, USA) were both applied in data analysis. To determine the prevalence of AF in patients with COVID-19, the exact binomial (Clopper–Pearson) method was used to calculate 95% confidence intervals (CIs). Estimates were standardized using the Freeman–Tukey double arcsine transformation. To elucidate the clinical impact of AF in patients with COVID-19, we pooled the crude odds ratios (ORs) for categorical outcomes using the inverse-variance method. The crude ORs were calculated by events and total numbers of patients in the AF groups and control groups. Moreover, we estimated the adjusted effect size by calculating the natural logarithm of the OR (log [OR]) and its standard error (SElog [OR]). The ORs were shown with 95% CIs. We evaluated the degree of heterogeneity among the included studies using the χ^**2**^ statistic (with a *P*-value of 0.10 considered significant) and the *I*^2^ test (25%, 50%, and 75% represent low, moderate, and high heterogeneity, respectively) (38). We used the random effect model in this study to improve the reliability of our results considering the potential heterogeneity.

Subgroup analyses were performed to research possible modulated factors influencing our primary meta-analysis results, including age, region, study design, sample size, cases of AF, and severity. We defined patients with severe COVID-19 as those who were admitted to the ICU, while patients who were not admitted to the ICU were considered to have non-severe COVID-19. Additionally, patients with a history of AF were excluded from the analysis of the prevalence of new-onset AF. Publication bias was assessed using funnel plots, Egger's test, and Begg's test. To appraise the robustness and reliability of the primary study outcomes, we also carried out sensitivity analyses by omitting each study in turn. All statistical tests were double-sided, and *P* < 0.05 was considered statistically significant.

## Results

### Literature Search

**Figure 1:**
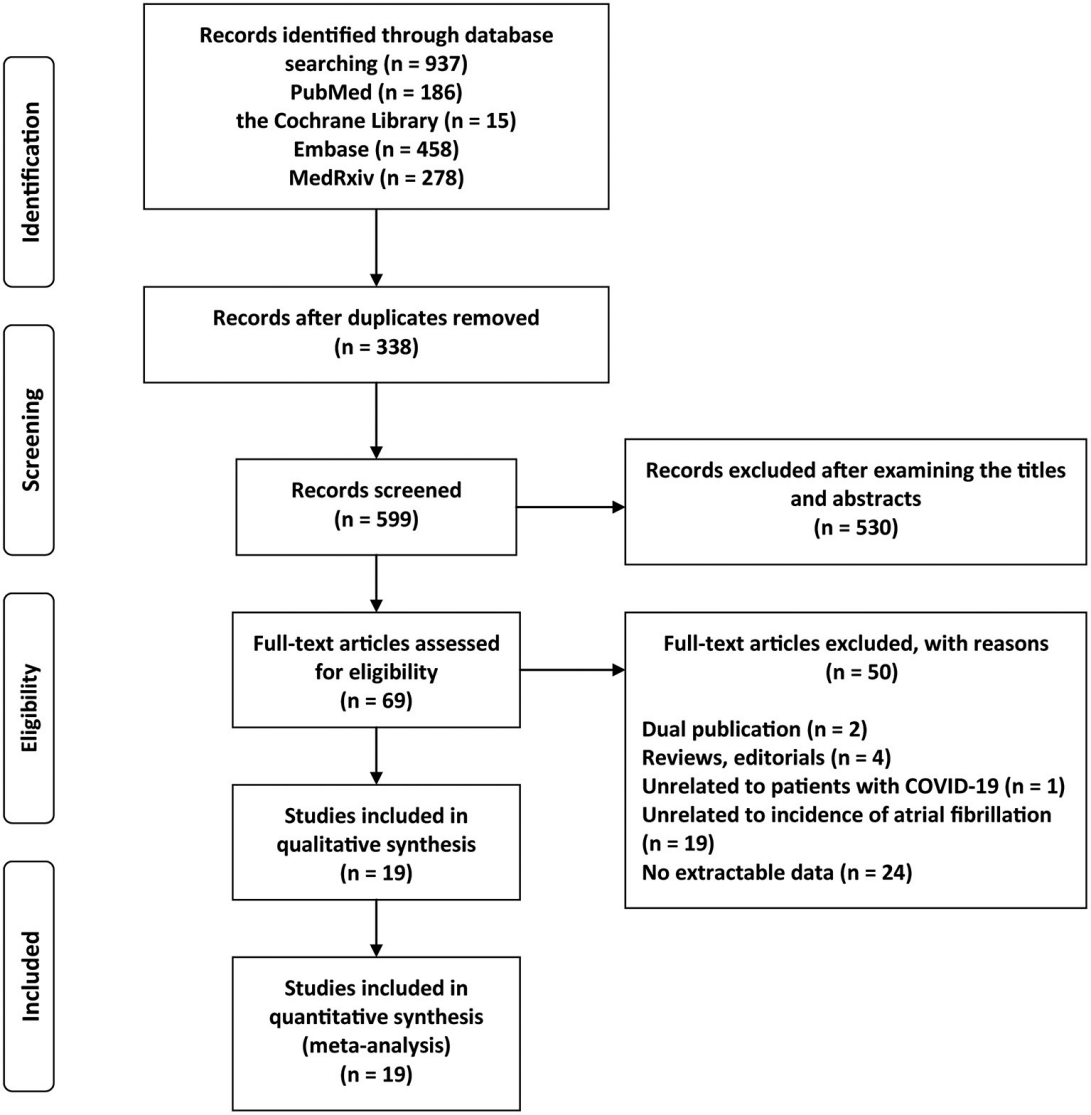
Flow chart of the study selection process.

The study selection process is shown in Figure 1. A total of 937 citations were identified through the initial database search. After a quick screening of the title and abstract, 69 articles remained. We further excluded 50 articles after the full-text review for the following reasons: (1) dual publication (*n* = 2); (2) editorials or review articles (*n* = 4); (3) unrelated to patients with COVID-19 (*n* = 1); (4) unrelated to the prevalence of AF (*n* = 19); and (5) no extractable data (*n* = 24). As a result, we included 19 eligible studies (14–32).

### Study Characteristics and Study Quality

The basic characteristics are shown in Table 1. Among the 19 included studies, (14–32) the publishing years ranged from 2020 to 2021. Overall, a total of 21,653 hospitalized patients were included, with 12,700 (58.7%) being men (ranging from 37.0 to 72.9%). The number of individuals ranged from 30 to 9,564, with the mean age of the participants ranging from 50.0 years to 75.2 years. Among the included studies, the diagnosis of AF was based on electrocardiograms. Three reports were from Asia, (16, 24, 25) 1 from Africa, (20) 7 from Europe, (17–19, 23, 26, 27, 29) and 8 from America (14, 15, 21, 22, 28, 30–32). Apart from 4 prospective cohort studies, (20, 22, 26, 27) the remaining 15 articles were designed as retrospective cohort studies (14–19, 21, 23–25, 28–32).

**Table 1:** Characteristics of included studies in this meta-analysis.

**References, country**	**Sample of size, N**	**AF diagnosis**	**Study design**	**Mean age (years), Male %**	**History of AF, N**	**AF cases, N**	**New-onset AF cases, N**	**Outcomes reported**	**Medication (%)**	**Adjustments**
Aajal et al. (20), Morocco	100	ECG	Prospective cohort	55.3, 37	22	2	NR	Prevalence	NR	–
Angeli et al. (23), Italy	50	ECG	Retrospective cohort	64, 72	NR	3	NR	Prevalence	Hydroxychloroquine: 82.0; Macrolides: 56.0; Lopinavir-Ritonavir: 54.0	–
Bhatla et al. (21), USA	700	ECG	Retrospective cohort	50, 45	39	25	NR	Prevalence, Mortality	Hydroxychloroquine: 24.6; Remdesivir: 8.1	None
Chen et al. (39), USA	143	ECG	Retrospective cohort	67, 62.2	19	13	13	Prevalence	NR	–
Colon et al. (14), USA	115	ECG	Retrospective cohort	56, 53.9	6	12	NR	Prevalence	Remdesivir/Placebo Trial: 7.0; Hydroxychloroquine: 6.1; Azithromycin: 43.5	–
Coromilas et al. (32), USA	4,526	ECG	Retrospective cohort	62.8, 57	408	509	NR	Prevalence	Hydroxychloroquine: 57.6; Azithromycin: 49.8; Antiviral: 15.3; IL-6 Inhibitor: 9.6; Anticoagulation: 29.4	–
Iacopino et al. (27), Italy	30	ECG	Prospective cohort	75.2, 66.7	8	10	10	Prevalence	None: 10.0; Antibiotic therapy: 6.7; Hydroxychloroquine+antiviral: 46.7; Hydroxychloroquine+ antiviral+azithromycin: 6.7; Hydroxychloroquine: 30.0; Monoclonal antibodies: 6.7; Low molecular weight heparins: 100.0	–
Kelesoglu et al. (25), Turkey	658	ECG	Retrospective cohort	54, 56.6	NR	33	33	Prevalence Mortality	NR	–
Linschoten et al. (18), Netherlands	3011	ECG	Retrospective cohort	67, 62.8	NR	142	NR	Prevalence	NR	–
Mountantonakis et al. (15), USA	9,564	ECG	Retrospective cohort	64.8, 58.9	687	1,687	1,109	Prevalence Mortality	NR	Matching for age, gender, smoking, race, medical history, lactate, WBC magnesium, procalcitonin,d-dimer, ferritin, CRP, creatinine, bun, AST, lymphocyte count, ALT, ALT phos, serum glucose, potassium, sodium
Oates et al. (31), USA	77	ECG	Retrospective cohort	69, 55	4	5	4	Prevalence	Hydroxychloroquine: 87.0; Azithromycin: 60.0; Remdesivir: 4.0; Tocilizumab: 4.0	–
Peltzer et al. (28), USA	1,053	ECG	Retrospective cohort	62, 62	94	166	101	Prevalence Mortality	Hydroxychloroqine: 70.8; Remdesivir: 4.9; Steroids: 22.9; IL-6 inhibitor: 6.2; Intravenous gamma globulin: 0.9	Age, sex, race, renal disease, hypoxia, heart failure, CAD, hypertension, diabetes, pulmonary disease, renal disease,immunosuppression, smoking status, and cancer
Rav-Acha et al. (16), Israel	390	ECG	Retrospective cohort	57.5, 55.4	21	20	16	Prevalence Mortality	Azithromycin: 24.2; Hydroxychloroquine: 37.9; QT prolonging drug: 17.2	None
Russo et al. (19), Italy	414	ECG	Retrospective cohort	66.9, 61.1	72	71	50	Prevalence Mortality	NR	None
Saleh et al. (22), USA	201	ECG	Prospective cohort	58.5, 57.2	14	17	17	Prevalence	Hydroxychloroquine/ Chloroquine: 40.8; (Hydroxychloroquine/ Chloroquine) + Azithromycin: 59.2	–
Sanz et al. (26), Spain	160	ECG	Prospective cohort	65.7, 60	30	12	12	Prevalence Mortality	NR	None
Wetterslev et al. (17), Denmark	155	ECG	Retrospective cohort	66, 72.9	NR	52	NR		NR	–
Yenercag et al. (24), Turkey	140	ECG	Retrospective cohort	51.7, 49.3	NR	13	NR	Prevalence	NR	–
Zylla et al. (29), Germany	166	ECG	Retrospective cohort	64.1, 65.1	NR	11	NR	Prevalence Mortality	Hydroxychloroquine: 44.6; Hydroxychloroquine + azithromycin: 16.3; Anticoagulation therapy: 30.7	None

*AF, atrial fibrillation; ECG, electrocardiogram; NR, not reported. WBC, white blood cell; CAD, coronary artery disease; CRP, C-reactive protein; BUN, blood urea nitrogen; AST, Aspartate aminotransferase, ALT, Alanine aminotransferase*.

Based on the Joanna Briggs Institute Critical Appraisal Checklist, all 19 studies (14–32) that reported the prevalence of AF met a minimum of six of the nine criteria, which meant that these articles applied rigorous methodology (Supplemental Table 2). In accordance with the NOS, all 8 studies (15, 16, 19, 21, 25, 26, 28, 29) that involved the association between AF and outcomes of patients with COVID-19 were viewed as moderate to high quality, with a score range of 6–8 (Supplemental Table 3).

### The Prevalence of AF in Patients With COVID-19

**Figure 2:**
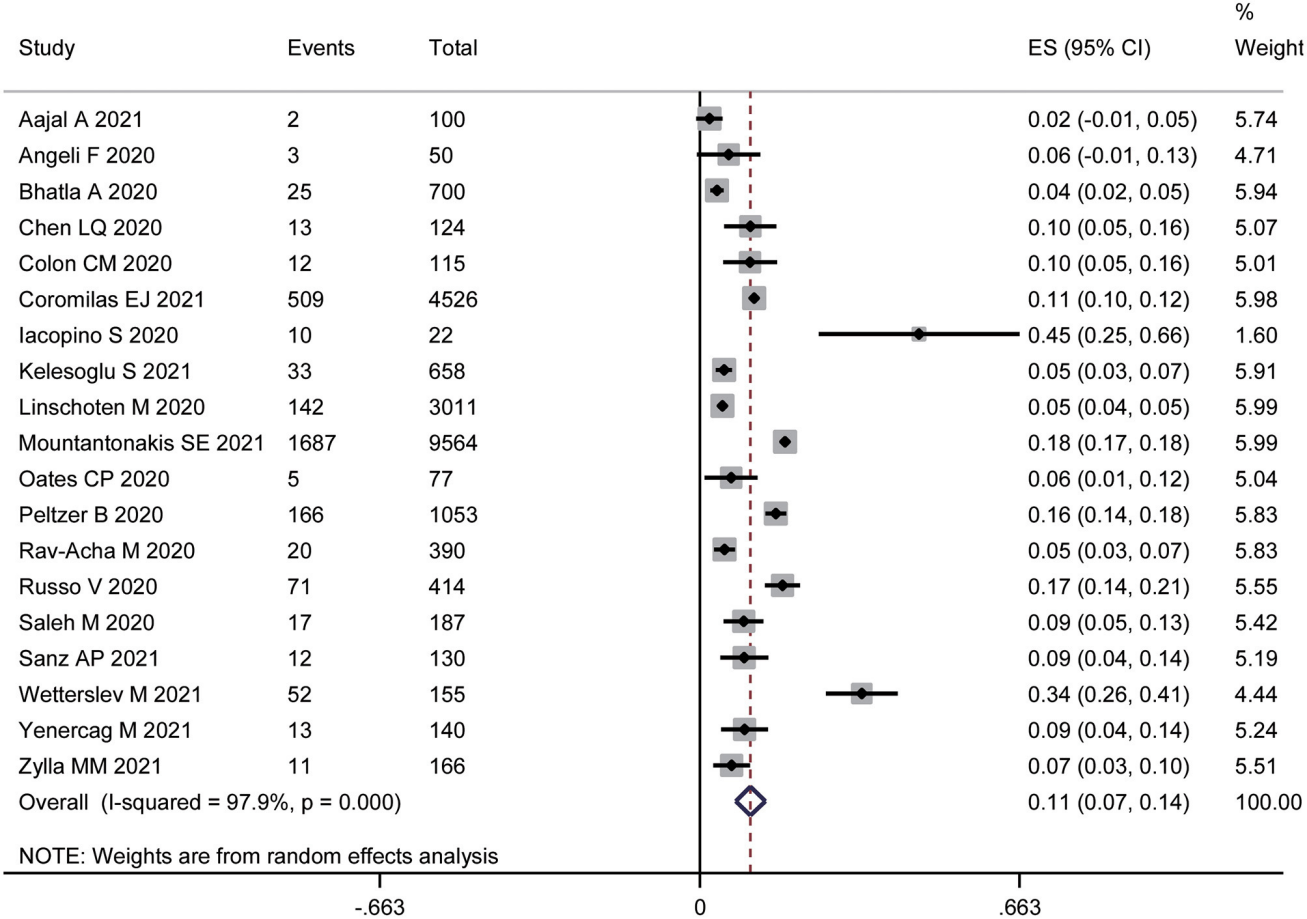
Forest plot of the prevalence of atrial fibrillation in patients with COVID-19.

Nineteen studies (14–32) with a total of 21,582 participants reported the prevalence of AF in hospitalized patients with COVID-19. The pooled prevalence of AF was 11% (95% CI: 7% to 14%), with high heterogeneity (I^2^ = 97.9%) (Figure 2).

**Figure 3:**
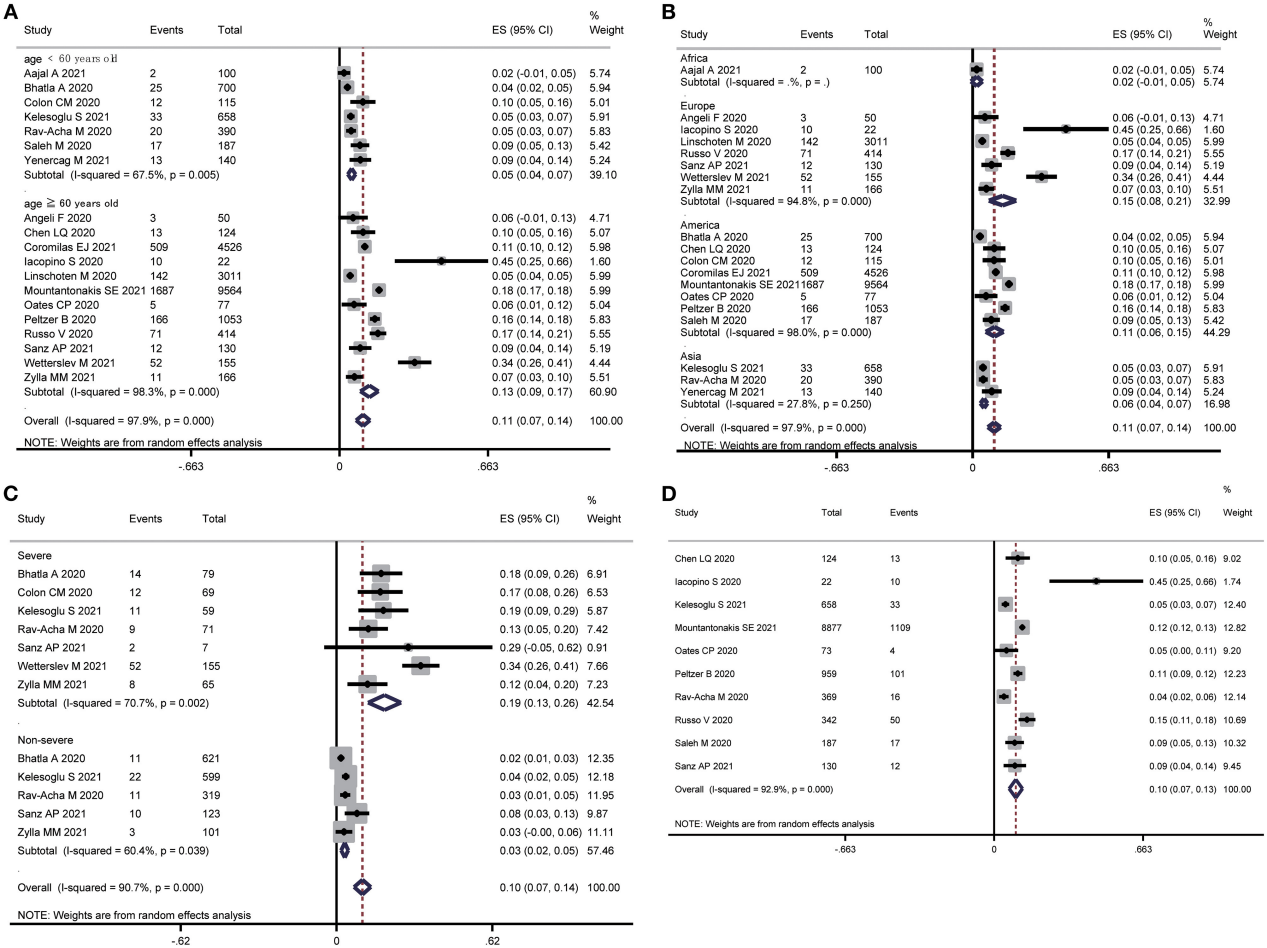
Subgroup analysis of the prevalence of atrial fibrillation in patients with COVID-19. **(A)** Age subgroup. **(B)** Region subgroup. **(C)** Severity subgroup. **(D)** New-onset atrial fibrillation subgroup.

In the subgroup analysis, older (mean age ≥60 years) patients with COVID-19 showed a nearly 2.5-fold higher prevalence of AF than younger (mean age <60 years) patients with COVID-19 (ES: 13 vs. 5%, *P* for subgroup difference < 0.001) (Figure 3A). Europeans had the highest prevalence of AF (ES: 15%), followed by Americans (ES: 11%), Asians (ES: 6%), and Africans (ES: 2%) (*P* for subgroup difference < 0.001) (Figure 3B). Furthermore, the prevalence of AF in patients with severe COVID-19 was 6-fold higher than in patients with nonsevere COVID-19 (ES: 19 vs. 3%, *P* for subgroup difference < 0.001) (Figure 3C). Ten articles (15, 16, 19, 22, 25–28, 30, 31) provided data on the prevalence of new-onset AF (ES: 10%, 95% CI: 7% to 13%, I^2^= 92.9%) (Figure 3D). There was no significant difference in the study design (*P* = 0.92), sample size (*P* = 0.74), or cases of AF (*P* = 0.20) (Table 2).

**Table 2:** Subgroup analysis of prevalence of AF in patient with COVID-19.

**Items**		**Number of studies**	**ES (95%CI)**	**P**	**P$P_{\text{h}(\%)}^{*}$**	**P^#^**
Result of primary analysis		19	0.105 (0.074–0.136)	<0.001	97.9	–
Mean age	<60 years	7	0.054 (0.037–0.072)	<0.001	67.5	<0.001
	≥60 years	12	0.133 (0.093–0.174)	<0.001	98.3	–
Study design	Retrospective	15	0.106 (0.072–0.140)	<0.001	98.3	0.92
	Prospective	4	0.102 (0.028–0.176)	0.007	88.2	–
Sample size	<300	11	0.110 (0.067–0.154)	<0.001	87.4	0.74
	≥ 300	8	0.100 (0.055–0.145)	<0.001	99.1	–
Cases of AF	<15	9	0.082 (0.049–0.116)	<0.001	73.6	0.20
	≥ 15	10	0.117 (0.076–0.159)	<0.001	98.9	–
Region	Europe	7	0.146 (0.080–0.212)	<0.001	94.8	<0.001
	America	8	0.107 (0.064–0.150)	<0.001	98.0	–
	Asia	3	0.055 (0.039–0.071)	<0.001	27.8	–
	Africa	1	0.020 (−0.007–0.047)	0.153	–	–
Severity of illness	Severe	7	0.191 (0.125–0.257)	<0.001	70.7	<0.001
	Non-severe	5	0.033 (0.018–0.047)	<0.001	60.4	–
Incidence of new-onset AF		10	0.097 (0.067–0.126)	<0.001	92.9	–

*AF, atrial fibrillation*.

^*^P for within-group heterogeneity,

# *P for subgroup difference*.

### The Impact of AF on All-Cause Mortality in Patients With COVID-19

**Figure 4:**
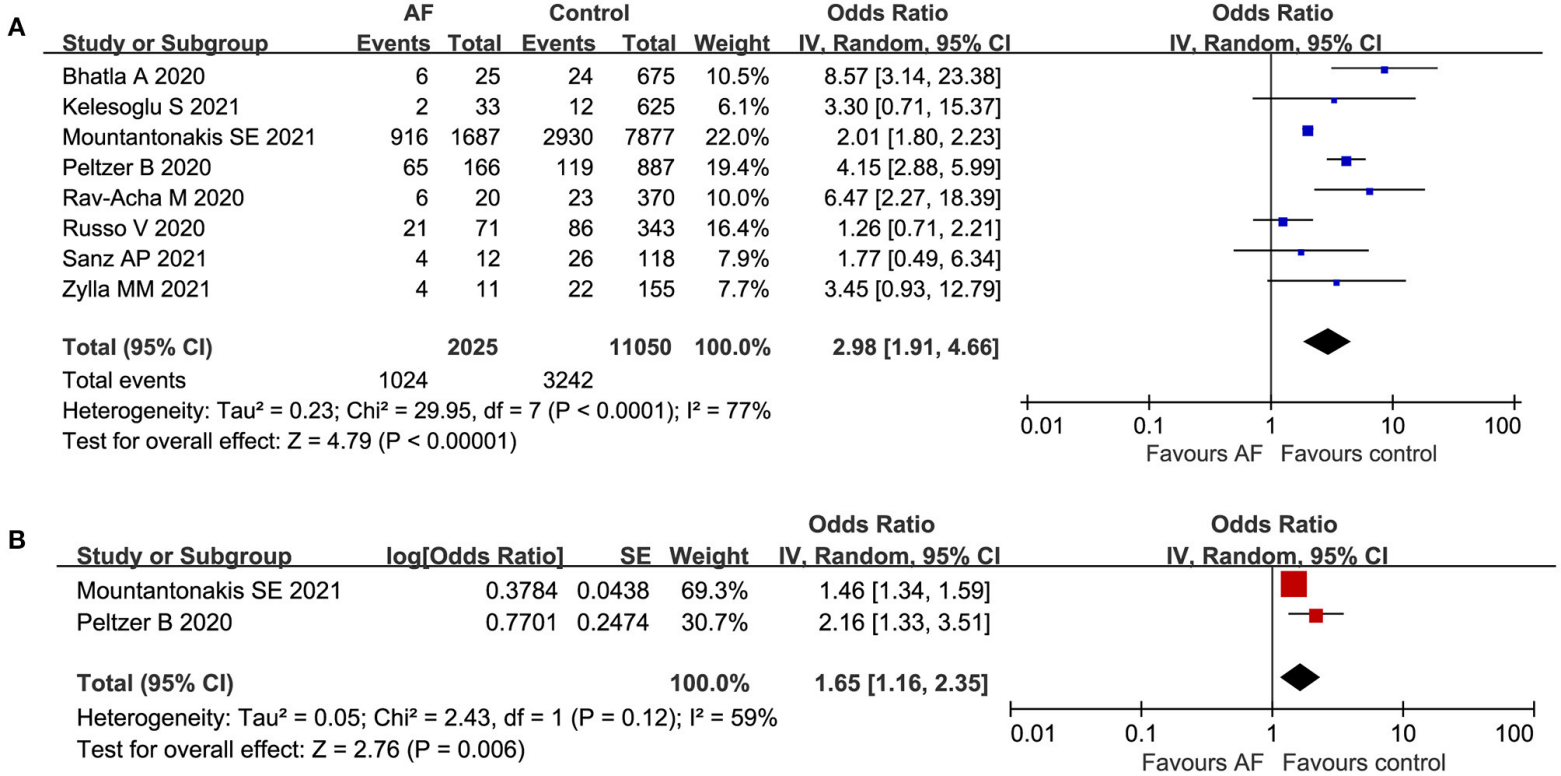
Forest plot of the association between atrial fibrillation and all-cause mortality in patients with COVID-19. **(A)** Crude effect size of the association between atrial fibrillation and all-cause mortality in patients with COVID-19. **(B)**. Adjusted effect size of the association between atrial fibrillation and all-cause mortality in patients with COVID-19.

Eight articles (15, 16, 19, 21, 25, 26, 28, 29) with a total of 13,075 participants reported the association between AF and all-cause mortality in patients with COVID-19. Ultimately, of the 2,025 patients in the AF group, 1,024 patients died (50.6%). There were 11,050 patients in the control group, with 3,242 deaths (29.3%). As presented in Figure 4A, AF was significantly associated with an increased risk of all-cause mortality among patients with COVID-19 (crude OR: 2.98, 95% CI: 1.91 to 4.66, I^2^= 77%). Moreover, the pooled result of the multivariate analysis (15, 28) did not change (adjusted OR: 1.65, 95% CI: 1.16 to 2.35, I^2^= 59%) (Figure 4B).

**Figure 5:**
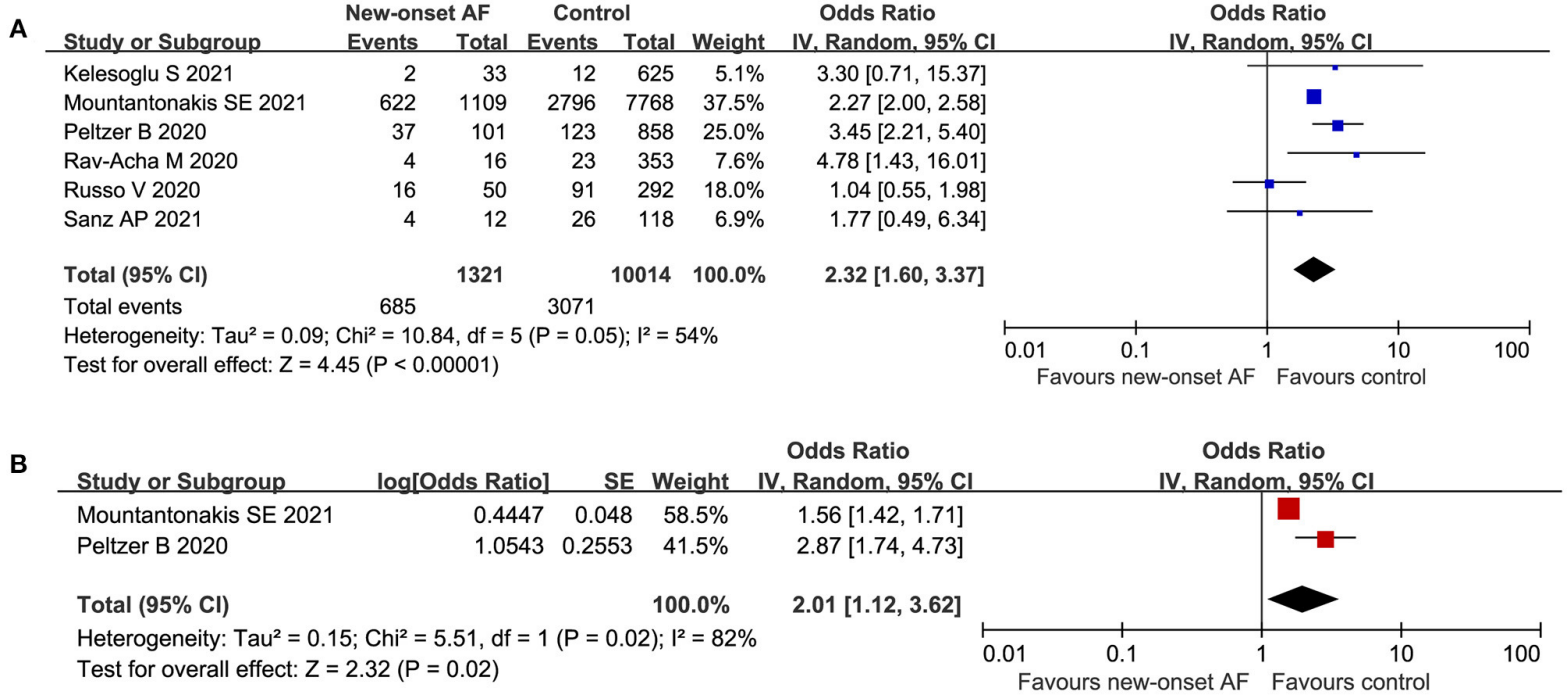
Forest plot of the association between new-onset atrial fibrillation and all-cause mortality in patients with COVID-19. **(A)** Crude effect size of the association between new-onset atrial fibrillation and all-cause mortality in patients with COVID-19. **(B)** Adjusted effect size of the association between new-onset atrial fibrillation and all-cause mortality in patients with COVID-19.

Additionally, 6 publications (15, 16, 19, 25, 26, 28) with a total of 11,335 participants reported the association between new-onset AF and all-cause mortality in patients with COVID-19. There was a strong association between new-onset AF and all-cause mortality among hospitalized patients with COVID-19 (crude OR: 2.32, 95% CI: 1.60 to 3.37, I^2^= 54%) (Figure 5A). Consistently, the pooled multivariate analysis (15, 28) showed similar results (adjusted OR: 2.01, 95% CI: 1.12 to 3.62, *P* = 0.02, I^2^= 82%) (Figure 5B).

As shown in Supplemental Figure 1, the funnel plot, Egger's test (*p* = 0.19), and Begg's test (*p* = 0.99) showed no statistically significant potential publication bias, although publication bias was not suggested when the included studies was limited (*N* <10). Sensitivity analyses performed by omitting each study indicated that our results were stable and reliable, with a range from 2.61 (95% CI: 1.69 to 4.02) to 3.55 (95% CI: 2.14 to 5.91) (Supplemental Figure 2).

## Discussion

Overall, 19 studies were included in this study with a total of 21,653 hospitalized patients. The pooled prevalence of AF approached 11% in patients with COVID-19. Our results demonstrated that AF is quite common among hospitalized patients with COVID-19, particularly among older patients (≥60 years of age), North American and European patients, and patients with severe COVID-19. Furthermore, AF and new-onset AF were significantly associated with an increased risk of all-cause mortality among hospitalized patients with COVID-19.

Our results seemed to agree with previous studies, (33–36) while there were essential differences between the present meta-analysis and others. Two previous meta-analyses (34, 35) did not specify the type of arrhythmia in COVID-19, which meant that those studies mainly focused on arrhythmias instead of each subtype, such as AF. Our meta-analysis extended these studies and had two important strengths. This is the most comprehensive study to assess the prevalence of AF, as well as new-onset AF, among hospitalized patients with COVID-19, and our results showed that new-onset AF was also independently associated with an increased risk of mortality by excluding data from patients with a prior history of AF. More importantly, our subgroup analyses first revealed regional differences in the prevalence of AF among hospitalized patients with COVID-19 and the correlation between AF and severe COVID-19.

Compared with the prevalence of arrhythmias in hospitalized patients with community-acquired pneumonia (7%, 95% CI: 6 to 9%), (40) this study showed a higher prevalence of AF in hospitalized patients with COVID-19 (11%, 95% CI: 7 to 14%). The exact pathophysiology underlying AF in COVID-19 may be multifactorial and remains elusive. At present, some studies preliminarily speculate that SARS-CoV-2 is similar to SARS-CoV, which may cause a series of cascade reactions leading to pneumonia by combining with angiotensin-converting enzyme-2 (ACE2) in the human respiratory tract and lung tissue (41). The ACE2 receptor is also widely expressed in the cardiovascular system (42). Theoretically, the cardiovascular system is also a potential target organ of SARS-CoV-2 (43). Therefore, ACE2-related signaling pathways may play a key role in myocardial injury, which may affect atrial remodeling and increase susceptibility to AF (44). Moreover, inflammatory factor storms may be the mechanism of disease progression (45). Various inflammatory factors have been proven to be closely related to the development of AF. It has been reported that even mild tension in rat atrial tissue pretreated with IL-6 can lead to the occurrence of AF (46). In addition to the direct damage to myocardial cells caused by virus infection and the systemic inflammatory response syndrome induced by the virus, metabolic abnormalities, (47) hypoxemia, (48–50) respiratory failure, and usage of certain antiviral drugs (51, 52) also play roles in the pathogenesis of AF.

The potential mechanism by which AF contributes to increased mortality in patients with COVID-19 is yet to be determined. Coagulation abnormalities, cardiac injury, and stroke are possible mechanisms. For example, patients with AF had marked elevations in troponin, brain natriuretic peptide, C-reactive protein, and D-dimer, which may be the manifestations of cardiac injury, worsening cardiac function, and inflammatory response (28). Furthermore, hypercoagulability is an important feature in COVID-19, and AF could contribute to poor cardiac output, exacerbate the hypercoagulable state, and eventually lead to increased mortality (53).

Our prognosis analysis showed that in-hospital mortality was significantly higher among patients with AF than among patients without AF. After adjustment for age, race, body mass index, and comorbidities, AF and new-onset AF were independently associated with a higher risk of all-cause mortality among patients with COVID-19. Moreover, it was notable that a few studies reported that new-onset AF was associated with longer hospital stays, more bleeding events, and more embolic events. These consistent findings indicated that AF and new-onset AF were associated with poor prognosis in patients with COVID-19. Therefore, clinicians should be more attentive to patients with COVID-19 and AF, optimize the clinical management of the disease, and implement more effective treatment regimens. Although no specific therapies have been recommended for patients with COVID-19 with AF to date, anticoagulant therapy may be useful. Systemic anticoagulants were reported to reduce mortality in hospitalized patients with COVID-19 (54). Similarly, low-molecular-weight heparin treatment was associated with lower 28-day mortality in patients with COVID-19 who had symptoms of coagulation disorders (55). In addition, several potential agents have been proposed for the treatment of patients with severe COVID-19, such as the interleukin-6 receptor antagonist tocilizumab (56, 57) and corticosteroids (58). Considering the strong link between inflammation and AF, the effect of these agents on the prevention of AF in patients with severe COVID-19 should be studied further.

Considering the high prevalence of AF among patients with COVID-19 and its poor prognostic implications, clinicians should recognize AF in patients with COVID-19. Careful electrocardiographic monitoring is advisable in patients with COVID-19 to detect AF early. Additionally, screening for AF should be performed in patients with COVID-19 and respective risk factors, particularly in older patients (≥60 years of age), North American and European patients, and patients with severe COVID-19. Moreover, our results highlight the importance of utilizing AF and new-onset AF as clinical markers of in-hospital mortality and poor prognosis in hospitalized patients with COVID-19. Future investigations will need to further explore the association between COVID-19 and AF and to evaluate the safest and most effective strategies for clinical treatment and management of the disease.

### Study Limitations

There are several limitations to the present systematic review and meta-analysis that need to be discussed. First, all the included studies were observational studies which cannot prove causality. Most of the studies were retrospective (79%) cohort. Hence, further well-designed, large-scale, prevalence studies are warranted to assessed the prevalence of AF in patient with COVID-19, as well as the potential difference in region, severity and age. Second, a high degree of heterogeneity was observed in our results. Although meta-regression was not performed, the subgroup analysis showed the heterogeneity might derived from region, age or severity (Table 2). Third, many studies did not adjust for clinical confounding factors regarding the outcome of death. However, the positive association between AF and all-cause mortality persisted in the adjusted subgroup, suggesting that our results were relatively stable. Fourth, all the included participants were inpatients, rather than community patients, which may overestimate the prevalence and clinical impact of AF on patients with COVID-19. Fifth, many articles did not report specific drugs for treatment, so we cannot address the effects of these factors on the association between AF and poor prognosis in patients with COVID-19. Sixth, it is well known that AF significantly contributes to the incidence of stroke; however, stroke was not assessed in the present meta-analysis. Nevertheless, studies have shown that stroke is an uncommon complication of COVID-19, and there is no significant association between cerebrovascular disease and fatal outcomes in patients with COVID-19, suggesting that the prognostic damage caused by AF might be independent of stroke (39, 59, 60). Finally, there was only a small number of studies from Asia and Africa. In light of varying population characteristics among different regions, more studies from Asia and Africa are needed to confirm the regional differences in the prevalence of COVID-19.

## Conclusions

AF is quite common among hospitalized patients with COVID-19, particularly among older patients (≥60 years of age), North American and European patients, and patients with severe COVID-19. Moreover, AF and new-onset AF were independently associated with an increased risk of all-cause mortality among hospitalized patients with COVID-19. Our results should be confirmed by further well-designed, prospective studies.

## Data Availability Statement

The original contributions presented in the study are included in the article/Supplementary Material, further inquiries can be directed to the corresponding author/s.

## Author Contributions

XL, WZ, and PY were responsible for the entire project and revised the draft. ZL and WS performed the study selection, data extraction, statistical analysis, and interpretation of the data. WS and XL drafted the first version of the manuscript. All authors participated in the interpretation of the results and prepared the final version of the manuscript.

## Funding

This work was supported by the National Natural Science Foundation of China (PY, 81760050 and 81760048; XL, 82100347) and the Jiangxi Provincial Natural Science Foundation for Youth Scientific Research (PY, 20192ACBL21037), as well as the Young Teachers' Basic Scientific Research Business Expenses Project (WZ, 20ykpy72), the China Postdoctoral Science Foundation (WZ, 2020M673016), and the China National Postdoctoral Program for Innovative Talents (WZ, BX20200400).

## Conflict of Interest

The authors declare that the research was conducted in the absence of any commercial or financial relationships that could be construed as a potential conflict of interest.

## Publisher's Note

All claims expressed in this article are solely those of the authors and do not necessarily represent those of their affiliated organizations, or those of the publisher, the editors and the reviewers. Any product that may be evaluated in this article, or claim that may be made by its manufacturer, is not guaranteed or endorsed by the publisher.
